# EGFR blockade in GBM brain tumor stem cells synergizes with JAK2/STAT3 pathway inhibition to abrogate compensatory mechanisms in vitro and in vivo

**DOI:** 10.1093/noajnl/vdaa020

**Published:** 2020-02-18

**Authors:** Katharine V Jensen, Xiaoguang Hao, Ahmed Aman, H Artee Luchman, Samuel Weiss

**Affiliations:** 1 Hotchkiss Brain Institute and Arnie Charbonneau Cancer Institute, Department of Cell Biology and Anatomy, Cumming School of Medicine, University of Calgary, Calgary, Alberta, Canada; 2 Drug Discovery Program, Ontario Institute for Cancer Research, Toronto, Ontario, Canada

**Keywords:** brain tumor stem cells, combinatorial strategies, glioblastoma, JAK2/STAT3

## Abstract

**Background:**

The EGFR pathway is frequently mutated in glioblastoma (GBM). However, to date, EGFR therapies have not demonstrated efficacy in clinical trials. Poor brain penetration of conventional inhibitors, lack of patient stratification for EGFR status, and mechanisms of resistance are likely responsible for the failure of EGFR-targeted therapy. We aimed to address these elements in a large panel of molecularly diverse patient-derived GBM brain tumor stem cells (BTSCs).

**Methods:**

In vitro growth inhibition and on-target efficacy of afatinib, pacritinib, or a combination were assessed by cell viability, neurosphere formation, cytotoxicity, limiting dilution assays, and western blotting. In vivo efficacy was assessed with mass spectrometry, immunohistochemistry, magnetic resonance imaging, and intracranial xenograft models.

**Results:**

We show that afatinib and pacritinib decreased BTSC growth and sphere-forming capacity in vitro. Combinations of the 2 drugs were synergistic and abrogated the activation of STAT3 signaling observed upon EGFR inhibition in vitro and in vivo. We further demonstrate that the brain-penetrant EGFR inhibitor, afatinib, improved survival in *EGFRvIII* mt orthotopic xenograft models. However, upregulation of the oncogenic STAT3 signaling pathway was observed following afatinib treatment. Combined inhibition with 2 clinically relevant drugs, afatinib and pacritinib, synergistically decreased BTSC viability and abrogated this compensatory mechanism of resistance to EGFR inhibition. A significant decrease in tumor burden in vivo was observed with the combinatorial treatment.

**Conclusions:**

These data demonstrate that brain-penetrant combinatorial therapies targeting the EGFR and STAT3 signaling pathways hold therapeutic promise for GBM.

**Key Points**

- Lack of efficacy of EGFR-targeted therapies in GBM is likely due to poor brain penetration of conventional inhibitors, lack of patient stratification for EGFR status, and mechanisms of resistance.
- Lack of efficacy of EGFR-targeted therapies in GBM.
- Combinatorial strategies targeting compensatory pathways activated in response to EGFR inhibition, such as STAT3 signaling, hold greater clinical potential for GBM.

**Importance of the Study**

Inhibition of promising druggable targets, such as EGFR, has failed to show efficacy in clinical trials for GBM. Poor brain penetration of many EGFR inhibitors and mechanisms of resistance, in response to EGFR inhibition, likely contribute to the failure of these therapies. We show here that the brain-penetrant EGFR inhibitor, afatinib, is effective at reducing BTSC growth in vitro and at extending survival in *EGFRvIII* mt orthotopic xenograft models. However, the STAT3 pathway becomes activated following afatinib treatment. Dual inhibition of EGFR and JAK2/STAT3 signaling, with the clinically relevant drugs afatinib and pacritinib, was effective at synergistically inhibiting BTSC growth in vitro and reducing tumor burden in vivo. These results show that combinatorial strategies targeting compensatory pathways activated in response to EGFR inhibition, such as STAT3 signaling, hold greater clinical potential for GBM.

Approximately 88% of glioblastoma (GBM) tumors have abnormalities in growth factor signaling, with alterations to the *EGFR* gene being particularly common and 40% of GBMs harboring *EGFR* amplifications, activating point mutations in the tyrosine kinase domain or the constitutively active *EGFRvIII* mutation.^1–3^ Given the prevalence of *EGFR* mutations in GBM, the development of therapeutics targeted to these aberrations has received considerable attention. In particular, *EGFRvIII* has attracted significant attention as a promising drug target. However, EGFR inhibitors, such as gefitinib and erlotinib, have failed to demonstrate more than modest results in most patients^4,5^ and, to date, anti-EGFR therapies have had little success in clinical trials (reviewed in Ref. ^6^).

The dearth of suitable blood brain penetrant compounds and the failure to stratify patients based on *EGFR* mutational status for clinical trials may have been some of the roadblocks for successful anti-EGFR therapies in GBM.^7^ Further, mechanisms of resistance, in response to EGFR inhibition, likely also contribute to the failure of these therapies.^8–12^ Upregulation of other pro-survival signaling pathways upon EGFR blockade have been proposed as likely mechanisms of resistance to EGFR inhibition in other cancers.^10,11,13^ Activation of STAT3 upon EGFR inhibition has been previously shown to lead to inadequate suppression of downstream targets, resistance to targeted drug therapies, and disease recurrence in head and neck and non-small-cell lung cancers (NSCLCs).^8,12,14,15^ STAT3 is a major regulator of tumorigenesis and is upregulated in a large subset of GBM.^16^ Previous studies by our group have shown that JAK2/STAT3 inhibition decreased brain tumor stem cell (BTSC) viability and improved the median overall survival in a BTSC orthotopic xenograft mouse model.^17,18^ EGFR and STAT3 signaling are exquisitely linked and it has been previously demonstrated that EGFRvIII can be phosphorylated by EGFRwt in GBM cells. One of the mechanisms for the subsequent phosphorylation of STAT3 requires EGFRvIII to undergo nuclear translocation and form an EGFRvIII–STAT3 nuclear complex.^19^ However, the signaling circuitry of EGFR in GBM is highly complex and there are additional signaling axes that have been reported to result in STAT3 activation.^8–12^

Given the relevance of both EGFR and STAT3 signaling in GBM tumorigenesis, here we further investigated STAT3 activation upon EGFR inhibition in GBM BTSCs. We used afatinib, a potent second-generation ErbB family blocker, that inhibits the activity of EGFR, HER, and ErbB4 and blocks trans-phosphorylation of ErbB3.^20^ Afatinib therapy is used to treat NSCLC and is part of a number of advanced trials for other lung cancers.^21^ Afatinib was tested in a phase I/II trial and demonstrated a manageable safety profile for recurrent GBM in combination with temozolomide (TMZ).^22^ In order to inhibit STAT3 signaling, we used pacritinib, a JAK2 inhibitor currently in phase III trials for myelofibrosis.^23^ We previously reported that pacritinib decreased BTSC viability in vitro and significantly increased overall median survival in combination with TMZ in mice orthotopically xenografted with an aggressive recurrent GBM BTSC culture.^18^

We report that concurrent inhibition of EGFR and JAK2/STAT3, with afatinib and pacritinib, abrogated the upregulation of STAT3 signaling seen upon EGFR inhibition in BTSCs. Combinatorial treatment was highly effective in a panel of molecularly heterogeneous BTSCs and in orthotopic *EGFRvIII* mt BTSC xenograft models. These results strongly suggest that targeting the EGFR and JAK2/STAT3 pathways in combination may be an effective therapeutic approach for the treatment of GBM.

## Methods

### Cell Culture and BTSC Culture Characterization

GBM BTSCs (*n* = 11) were cultured from tumor specimens as previously described.^24^ Normal human astrocytes (Lonza) were cultured according to the manufacturer’s instructions. For viability assays, 500–1000 BTSCs per well were treated with vehicle (DMSO) or drug and alamarBlue conversion was measured 7–14 days later. For neurosphere assays, 500–1000 BTSCs per well were treated with vehicle or drug and the number of spheres formed was counted 7–21 days later. The BTSC cultures used in the study have been previously characterized for mutational status using RNAseq and protein analysis as well as whole-genome sequencing in a number of prior studies.^17,18,24–28^

### Limiting Dilution Analysis

Cells were plated at decreasing densities (512 cells per well to 1 cell per well) and treated with vehicle (DMSO), afatinib (Selleck Chemicals), pacritinib (CTI BioPharma), or a combination. After 21 days, the number of wells that established sphere(s) were counted. The Extreme Limiting Dilution Analysis computational program^29^ was used to analyze the data.

### Cytotoxicity Assays

Cells were plated as described above and treated with vehicle (DMSO) or drug. YOYO-1 (Essen BioScience) was used to determine the cell death index, which was calculated by taking the ratio of green fluorescent surface area to total cell surface area using the Incucyte Zoom Live Imaging System (Essence BioScience).

### Immunoblotting

Western blotting was performed using standard protocols. Primary antibodies included p-STAT3 Y705 (1:1000; Cell Signaling Technology [CST]), STAT3 (1:1000; CST), p-EGFR Y1068 (1:1000; CST), EGFR (1:1000; CST), p-p44/42 MAPK (T202/Y204) (1:1000; CST), p44/42 MAPK (1:4000; CST), β-tubulin (1:1000; CST), and Actin (1:1000; Santa Cruz Biotechnology). Western blots were quantified using standard densitometry protocols with ImageJ.

### Bliss Independence and Excess Over Bliss Synergy Analyses

BTSCs were treated with suboptimal doses of afatinib, pacritinib, or a combination. Bliss independence analyses were performed as previously described.^18,29^ Further synergy studies were conducted in a 4 × 7 plating matrix where BTSC cells were treated with pacritinib (0.1–1 μM) and afatinib (1–150 nM) concurrently. The “Excess over Bliss” is the difference between the predicted combination response and the experimentally observed combination response.

### Pharmacokinetic Analyses

Nontumor-bearing mice (*n* = 3) were treated with vehicle (0.5% w/v methylcellulose), 15 mg/kg, or 30 mg/kg afatinib by oral gavage for 5 consecutive days. On day 5, blood was collected at 30 and 300 min and brains were harvested at 300 min following treatment. Afatinib (15 mg/kg) and pacritinib (100 mg/kg) were also administered concurrently to assess brain penetration when the drugs were co-administered as described above. Liquid chromatography–mass spectrometry was performed to determine the serum and brain concentration of afatinib and pacritinib.

### Intracranial BTSC Xenografts

All animal procedures were performed according to our animal ethics protocol (AC17-0215), approved by the Animal Care Committee of the University of Calgary and operating under the Guidelines of the Canadian Council on Animal Care. BT67 (*EGFR* wt) (100 000 cells), BT73, and BT147 (*EGFRvIII* mt) (50 000 cells) were stereotactically implanted into the brains of SCID mice, as previously described^17,18^ for pharmacodynamic (PD) analysis or to assess survival. For PD studies, 2 weeks post-xenograft of BT73 (*EGFRvIII* mt) cells, mice were treated for 5 consecutive days as follows: vehicle (0.5% w/v methylcellulose in ddH_2_O), pacritinib (100 mg/kg), afatinib (15 mg/kg), or a combination. Two hours following the final treatment, animals were euthanized, and brains were processed for immunohistochemistry.

Kaplan–Meier survival studies were performed as previously described.^18^ Briefly, BT67 (*EGFR* wt), BT73 (*EGFRvIII* mt), or BT147 (*EGFRvIII* mt) tumor-bearing mice were randomized to vehicle or treatment cohorts 1 week following xenografts. Vehicle (0.5% w/v methylcellulose) or 15 mg/kg afatinib were administered via oral gavage. A 3-week regimen of 5 weekly treatments was used for BT73 (*EGFRvIII* mt), a 6-week regimen of thrice weekly treatments for BT147 (*EGFRvIII* mt), and an 8-week regimen of once weekly treatment followed by an 11-week regimen of thrice weekly treatments was used for BT67 (*EGFR* wt). Different treatment regimens were designed based on the different survival times of each of the BTSC mouse xenograft models. For all Kaplan–Meier studies, a minimum of 9 mice were used per treatment cohort.

### Immunohistochemistry

Tissue sections were incubated in primary antibodies, including anti-human nucleolin (Abcam), p-EGFR Y1068 (1:200; CST), and p-STAT3 Y705 (1:40; CST) overnight, followed by secondary antibodies, including biotin-conjugated goat anti-mouse and goat anti-rabbit (Jackson ImmunoResearch). Vectastain Elite kits (Vector Laboratories) were used for detection.

### Magnetic Resonance Imaging

T2-weighted imaging was performed using a 9.4-T Bruker horizontal-bore magnetic resonance (MR) system. Tumor burden was quantified using ImageJ software. The tumor was outlined in each of the 12 0.5 mm serial sections obtained using MR imaging. The volume of each section was calculated and summed to obtain the total tumor volume. Matching nucleolin staining was used in parallel to validate the area that was outlined for quantification.

### Microscopy

Images of BTSCs were captured using a Zeiss Axiovert 40 CFL inverted microscope and AxioVision software. The Incucyte Zoom Live Imaging System (Essence Bioscience) was used for cytotoxicity assays and to image BTSCs. An Olympus Slide Scanner was used to image the brain sections. OlyVIA (Olympus) software was used to analyze the images.

### Statistical Analyses

Data are illustrated in bar graphs, including mean ± SD, *n* ≥ 3 biological replicates, and 3–6 technical replicates. Asterisks denote statistical significance. For Kaplan–Meier studies, the statistical difference in median survival was determined by the log-rank test. For MRI studies, the statistical difference in tumor burden was calculated using unpaired *t*-tests corrected for multiple comparisons using the Holm-Sidak method.

## Results

### On-target Inhibition of EGFR Signaling With Afatinib Decreases BTSC Viability and Sphere Formation

**Fig. 1:**
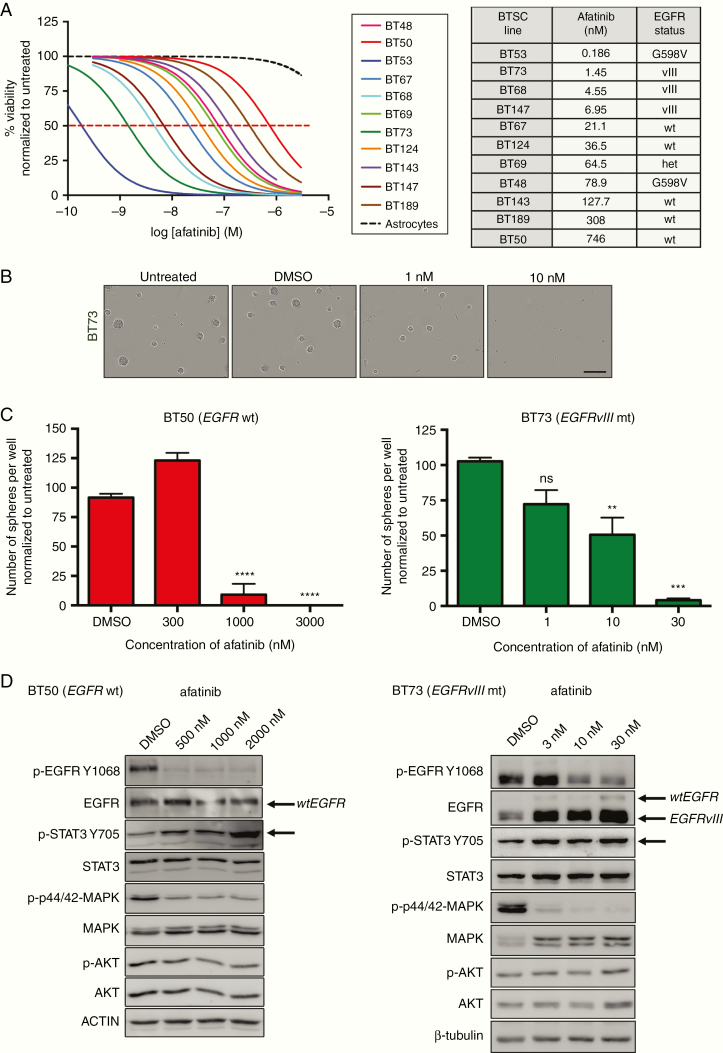
Afatinib effectively decreases BTSC viability and sphere-forming capacity and has on-target activity on phospho-EGFR. (A) Afatinib decreased cell viability in molecularly diverse BTSCs. Normal human astrocytes were unaffected. (B) Representative images of BT73 (*EGFRvIII* mt) spheres following afatinib treatment. The scale bar represents 300 μm. (C) Quantification for 2 representative BTSC cultures, BT50 (*EGFR* wt) and BT73 (*EGFRvIII* mt) (*P < .05, **P < .01, ***P < .001, and ****P < .0001 vs DMSO; Sidak’s multiple comparison two-way ANOVA). Error bars represent SD. (D) Afatinib demonstrates on-target activity as seen by a decrease in p-EGFR Y1068 levels. An increase in p-STAT3 Y705 levels was observed (representative BTSC cultures, BT50 [*EGFR* wt] and BT73 [*EGFRvIII* mt] shown).

We first asked whether the EGFR inhibitor afatinib could reduce BTSC viability and sphere formation, using alamarBlue and neurosphere assays. Afatinib inhibited BTSC growth and sphere-forming capacity in a dose-dependent manner at nanomolar concentrations, while normal human astrocytes were unaffected (Figure 1A–C). Afatinib-mediated EGFR inhibition was effective against all BTSCs tested; however, the degree of sensitivity was influenced by the status of common GBM molecular alterations, including *EGFR* mutations and *PTEN* inactivation (Figure 1A and Supplementary Table S1). BT53, an *EGFR*-mutant BTSC culture with an activating G598V mutation and the *EGFRvIII* mt BTSC cultures, BT73, BT68, and BT147 responded to afatinib treatment at low nanomolar concentrations and were more sensitive to afatinib than the *EGFR-*wildtype BTSC cultures (Figure 1A). This was also observed with 2 other EGFR inhibitors tested, erlotinib (Supplementary Figure S1A and B) and AZD9291 (Supplementary Figure S1C and D).

Afatinib demonstrated on-target activity as seen by the decrease in p-EGFR Y1068 in both *EGFR* wt and *EGFRvIII* mt BTSCs (Figure 1D and Supplementary Figure S1E). Further, afatinib-treated BTSCs showed decreased levels of p-p44/42 MAPK (T202/Y204), a downstream effector of the EGFR pathway. Interestingly, EGFR inhibition with afatinib led to increased levels of p-STAT3 Y705 in both BT50 (*EGFR* wt) and BT73 (*EGFRvIII* mt) (Figure 1D and Supplementary Figure S1E), but not in p-AKT or p-MAPK (through a TNF activation response), which have also been previously described as possible mechanisms of resistance following EGFR inhibition in lung cancer^30^ and glioma,^31^ respectively (Figure 1D). These data thus highlight a potential compensatory mechanism in response to EGFR inhibition, in BTSCs, specifically through activation of STAT3.

### Combined Inhibition of the EGFR and JAK2/STAT3 Pathways Is Effective in BTSCs

We next asked whether concurrent inhibition of EGFR and JAK2/STAT3 signaling would be more effective than inhibition of either pathway alone. We tested 4 EGFR inhibitors in combination with pacritinib (Supplementary Figure S2). All the EGFR inhibitors tested displayed efficacy when combined with pacritinib (Supplementary Figure S2). Afatinib was used for further combinatorial investigations as it is the most clinically relevant drug.

**Fig. 2:**
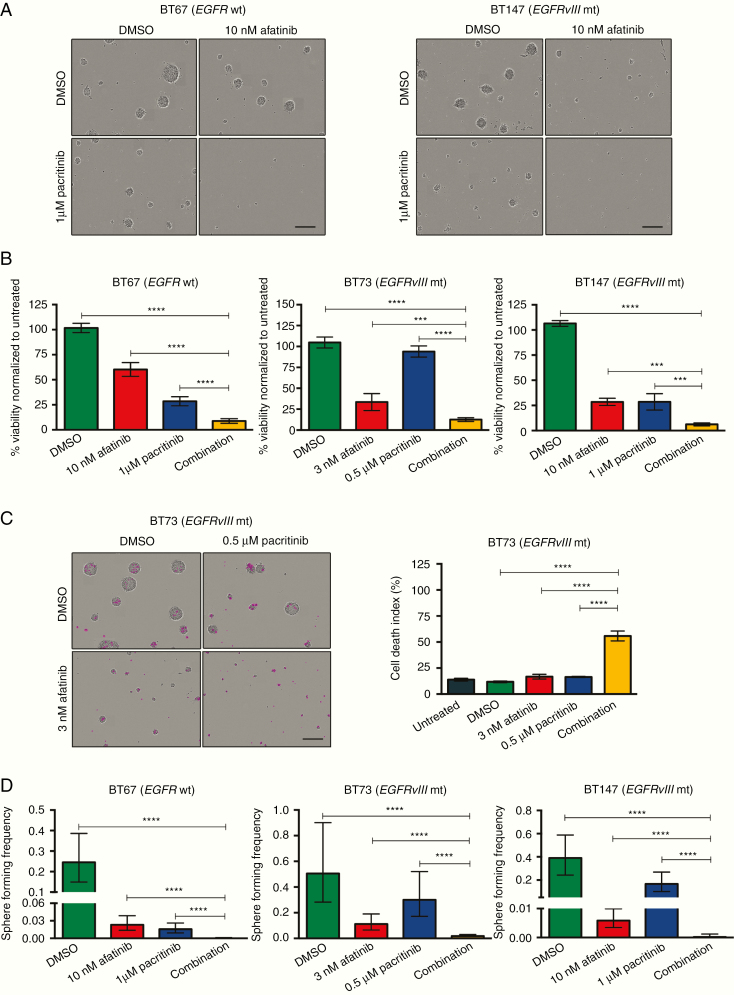
Combined inhibition of the EGFR and JAK2/STAT3 pathways is more effective than inhibition of either pathway alone. (A) Combined treatment with afatinib and pacritinib decreased sphere formation. Representative images for BT67 (*EGFR* wt) and BT147 (*EGFRvIII* mt) shown. The scale bar represents 300 μm. (B) Combined treatment decreased alamarBlue conversion (***P < .001 and ****P < .0001 vs DMSO; Sidak’s multiple comparison two-way ANOVA) in 3 representative BTSC cultures, BT67 (EGFR wt), BT73 (EGFRvIII mt), and BT147 (EGFRvIII mt). (C) Representative images for BT73 shown. The scale bar represents 300 μm. Quantification of cell death index (****P < .0001; Sidak’s multiple comparison two-way ANOVA). (D) Combined inhibition of BTSCs results in a significant decrease in sphere-forming frequency compared to the inhibition of a single pathway (****P < .0001).

We used suboptimal doses of the JAK2 inhibitor pacritinib in combination with suboptimal doses of afatinib for 7–21 days. The combined treatment effectively decreased viability in all BTSCs tested (Figure 2A and B and Supplementary Figure S3A). Furthermore, combined treatment dramatically increased cell death (Figure 2C and Supplementary Figure S3B, representative images are shown). Next, using limiting dilution assays, we found that while both afatinib and pacritinib decreased the sphere-forming frequency of BTSCs, sphere formation was fully abrogated in the combination treatment (Figure 2D and Supplementary Figure S3C).

It would also be of high clinical relevance to further investigate other JAK/STAT3 inhibitors, existing or currently under development, in combinatorial studies with brain-penetrant EGFR inhibitors such as afatinib. We show here that the JAK2/STAT3 inhibitor WP1066, when combined with afatinib, also effectively decreased BTSC viability and sphere formation (Supplementary Figure S4A).

### Combined Inhibition of the EGFR and JAK2/STAT3 Pathways Is Synergistic in BTSCs

**Fig. 3:**
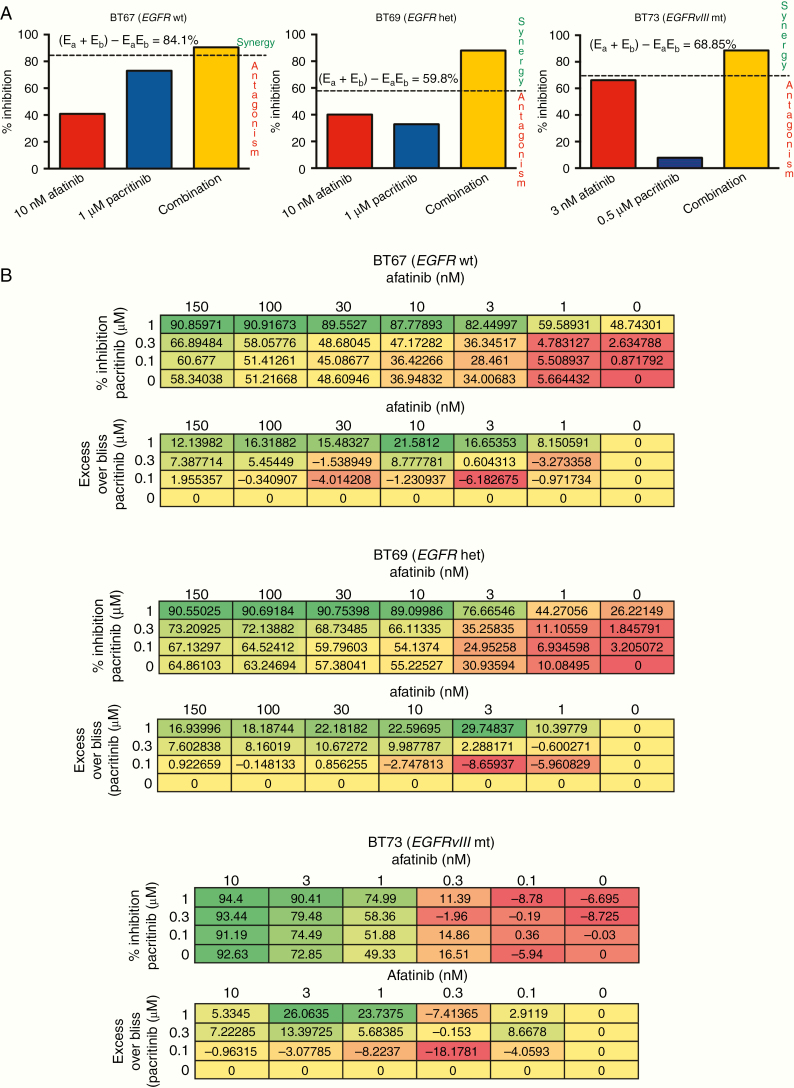
Combined inhibition of the EGFR and JAK2/STAT3 pathways is synergistic in BTSCs. Representative BTSCs BT67 (*EGFR* wt), BT69 (*EGFR* het), and BT73 (*EGFRvIII* mt) are shown. (A) Combined treatment with suboptimal doses of afatinib and pacritinib was synergistic as shown by bliss independence in BTSCs. (B) Percent inhibition and excess over bliss values are shown for 3 representative BTSCs. An excess over bliss greater than 0 indicates synergy. Excess over bliss equal to 0 indicates additive. Excess over bliss less than 0 indicates less than additive.

We next asked whether afatinib and pacritinib were synergistic in inhibiting BTSC growth. Synergism is observed when the combined effect of the 2 drugs is greater than that predicted by their individual effects. Interestingly, a combination of suboptimal doses of afatinib and pacritinib displayed synergy in all of the BTSCs tested (Figure 3A and Supplementary Figure S4B). Synergy was observed with a combination effect above the calculated bliss expectation (*E* = (*A* + *B*) − (*A* × *B*)), where A and B are the fractional growth inhibitions of drug A (afatinib) and B (pacritinib) at a given dose. For all BTSCs tested, the combination effect was above the calculated bliss expectation indicating synergy (Figure 3A and Supplementary Figure S4B).

We further assessed synergy between afatinib and pacritinib with a more stringent quantitative approach using multiple-dose combinations. We used an excess over bliss analysis^32^ to examine a larger number of combinations (Figure 3B). In BT67 (*EGFR* wt), BT69 (*EGFR* het), and BT73 (*EGFRvIII* mt), extensive synergy was observed when pacritinib and afatinib were used in combination, as demonstrated by excess over bliss values greater than 0 (Figure 3B). Extensive synergy was also observed when using erlotinib in combination with pacritinib (Supplementary Figure S4C) in both *EGFR* wt and *EGFR* mt BTSCs.

### Systemic Administration of Afatinib Inhibits Intracranial EGFR Signaling and Provides a Survival Advantage for Mice Xenografted With *EGFR*-Mutant BTSCs

**Fig. 4:**
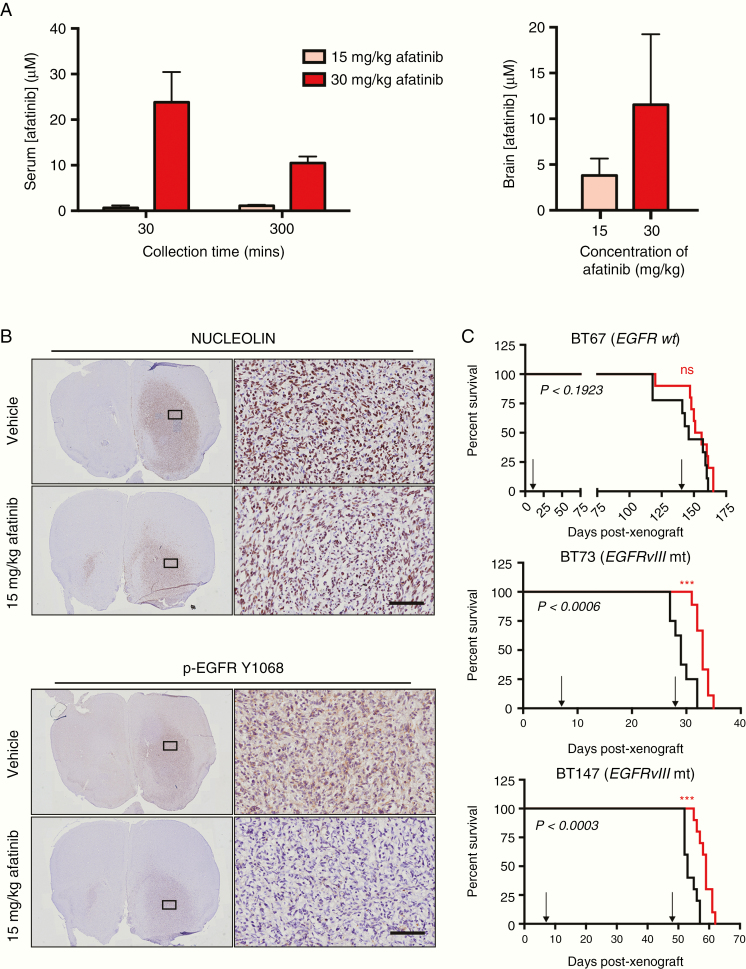
Afatinib accumulates in the plasma and brains of SCID mice, demonstrates on-target activity, and improved median survival in *EGFR*-mutant orthotopic BTSC mouse models. (A) Serum concentration of afatinib at 30- and 300-min post-dosing with 15 or 30 mg/kg of afatinib. Brain concentration of afatinib at 300 min post-dosing. Values represent mean ± SD. (B) Large tumors were visualized in both the vehicle and afatinib-treated mice using anti-human nucleolin staining. Five consecutive days of treatment, following tumor establishment, with 15 mg/kg of afatinib inhibited EGFR activation in BT147 (*EGFRvIII* mt) orthotopic xenografts. Scale bars represent 100 μm and apply to all of the higher magnification images. (C) 15 mg/kg afatinib failed to provide a survival advantage in *EGFR* wt BT67 (*n* = 10 mice per group, *P* < .1923; log-rank test) but provided a significant survival advantage in BT73 (*EGFRvIII* mt) (*n* = 9 mice per group, *P* < .0006; log-rank test) and BT147 (*EGFRvIII* mt) (*n* = 10 mice per group, *P* < .0003; log-rank test). Arrows show the start and end of the treatment period for each Kaplan–Meier curve.

Afatinib has been shown to penetrate the blood-brain barrier (BBB) in intracerebral metastases of NSCLC.^33^ We also found that afatinib accumulated at micromolar concentrations in a nonlinear manner in brains of SCID mice (Figure 4A). Mice bearing intracranial BT147 (*EGFRvIII* mt) xenograft tumors were divided into 2 cohorts: vehicle and 15 mg/kg of afatinib. Anti-human nucleolin staining confirmed tumor establishment 4 weeks post-xenografts (Figure 4B). Five consecutive days of treatment with afatinib effectively inhibited intracranial EGFR signaling as seen by a reduction in the positive staining for p-EGFR Y1068 (Figure 4B).

We next investigated whether afatinib could provide a survival advantage. Treatment with 15 mg/kg afatinib failed to provide a survival advantage in mice bearing tumors from BT67 (*EGFR* wt) (Figure 4C). However, a significant survival advantage was observed for mice xenografted with either BT73 or BT147, *EGFRvIII* mt BTSCs (Figure 4C). The differential response to EGFR inhibition in these 3 BTSC xenograft models highlights the importance of the *EGFR* mutational status with regard to response to EGFR-targeted therapies.

### Concurrent Systemic Administration of Afatinib and Pacritinib Shows Inhibition of EGFR and JAK2/STAT3 Signaling in Orthotopic BTSC Xenografts

**Fig. 5:**
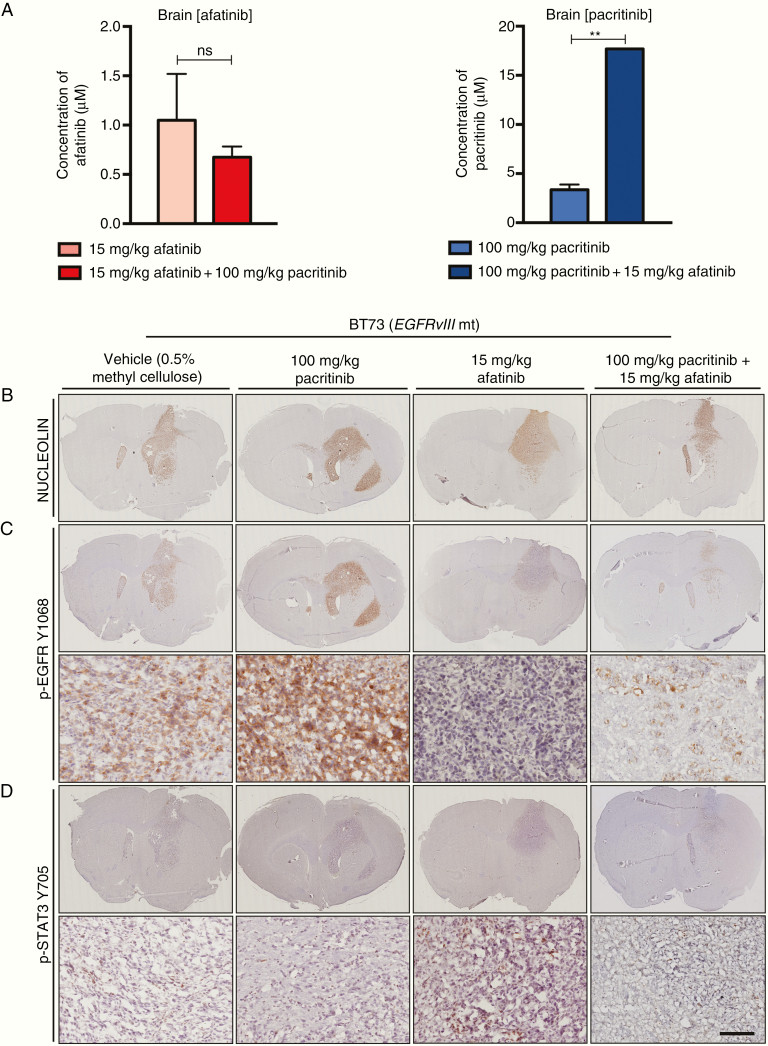
Afatinib and pacritinib effectively penetrate the brain and demonstrate on-target activity in orthotopic BTSC xenografts. (A) Brain concentrations of afatinib and pacritinib at 300 min post-dosing with single agents or concurrent administration. Values represent mean ± SD (**P < .0014; unpaired *t*-test). (B) Anti-human nucleolin was used to confirm the presence of tumor burden. (C) A decrease in positive p-EGFR Y1068 staining was seen following 15 mg/kg treatment of afatinib. There was a further decrease in positive p-EGFR Y1068 staining for the combination-treated group. (D) An increase in positive p-STAT3 Y705 signal was seen in the 15 mg/kg afatinib treated group. The increased activation of STAT3 was abolished in the combination group. The scale bar represents 100 μm and applies to all of the higher magnification images.

We next asked whether afatinib and pacritinib would effectively inhibit intracranial EGFR and JAK2/STAT3 signaling. Afatinib and pacritinib were concurrently administered for 5 consecutive days. The concentrations of afatinib in serum and brain did not change with the combination treatment, compared to afatinib alone. However, the pacritinib concentration in the brain increased approximately 5-fold over pacritinib alone when co-administered with afatinib (Figure 5A). While the mechanism of action and implications of this increased accumulation will need to be investigated in future studies, these data show that both afatinib and pacrtinib still effectively penetrate the BBB when delivered concomitantly.

We xenografted BT73 (*EGFRvIII* mt) cells in SCID mice. Two weeks following xenografts, mice were randomized into 4 treatment cohorts: vehicle, 15 mg/kg afatinib, 100 mg/kg pacritinib, or a combination of 15 mg/kg afatinib and 100 mg/kg of pacritinib. Mice were sacrificed following 5 consecutive days of treatment. Anti-human nucleolin immunostaining confirmed tumor establishment (Figure 5B). Afatinib effectively inhibited intracranial EGFR signaling as seen by a dramatic reduction in the positive staining for p-EGFR Y1068 (Figure 5C). Furthermore, stronger staining of p-STAT3 Y705, in particular at the infiltrating edges of tumors, was observed in the afatinib-treated group compared to the other treatment cohorts (Figure 5D). The intracranial increase in p-STAT3 Y705 upon EGFR inhibition was decreased in the mice that received concomitant treatment with afatinib and pacritinib (Figure 5D).

### Concomitant Treatment With Afatinib and Pacritinib Results in Decreased Tumor Growth

We next assessed the efficacy of combinatorial treatment in vivo. Mice xenografted with BT73 (*EGFRvIII* mt) cells were treated with vehicle, 15 mg/kg of afatinib, 100 mg/kg of pacritinib, or a combination of afatinib and pacritinib. The afatinib arm showed survival benefit over the control and pacritinib only arms (Supplementary Figure S5A). However, there were symptoms of toxicity upon prolonged treatment with the 2 drugs in the combinatorial arm, which were not observed in the vehicle-treated or single-arm groups. The animals in the combinatorial group displayed lethargy, weight loss, and some neurotoxicity including limb paralysis. Mice in this group were sacrificed based on the humane endpoints according to animal health care guidelines. Therefore, no significant improvement in survival was observed in the combinatorial study arm compared toafatinib alone (Supplementary Figure S5A). However, animals in the combinatorial arm did not appear to display symptoms associated with high tumor burden.

**Fig. 6:**
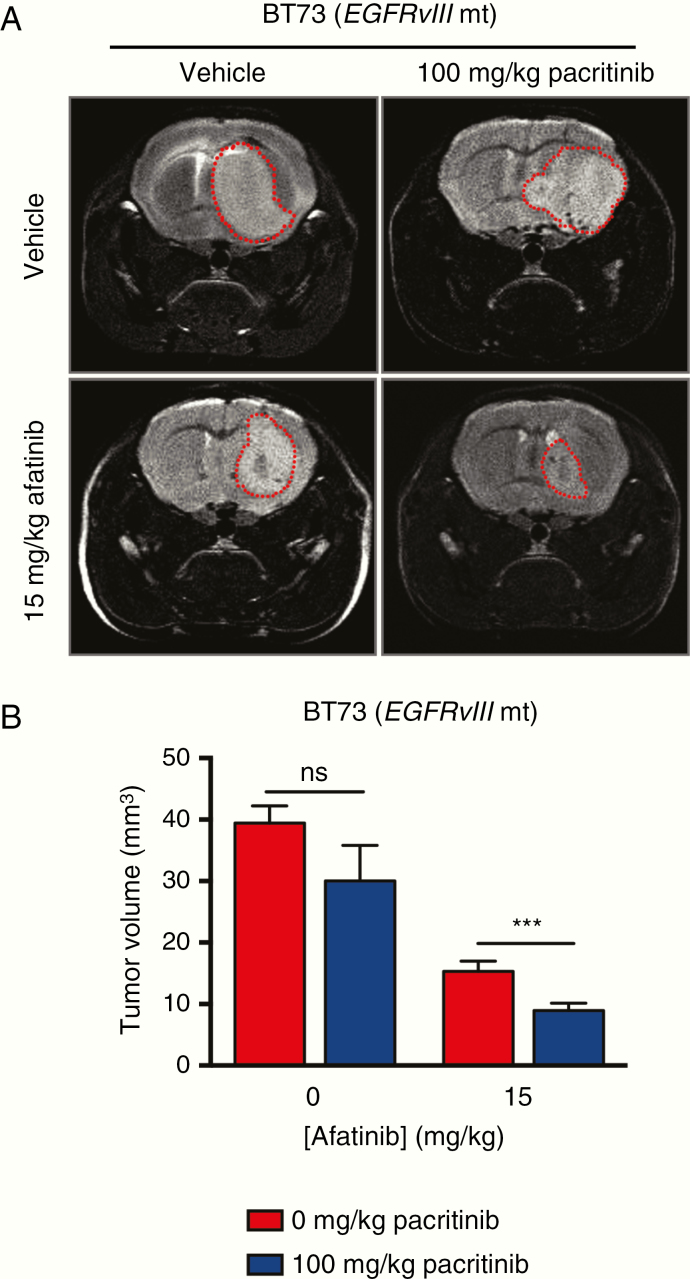
Systemic administration of afatinib and pacritinib decreases tumor burden in *EGFRvIII* mt orthotopic BT73 xenografts. (A) Representative MR images for the 4 different treatment groups following 3 weeks of treatment. (B) Quantification of tumor volume for the different treatment groups following 3 weeks of treatment. Statistical significance was determined using unpaired *t*-tests corrected for multiple comparisons using the Holm-Sidak method (***P < .0021). Error bars represent SD.

We therefore proceeded to analyze tumor burden by MR imaging, as described in the Methods section, following 3 consecutive weeks of dosing. The tumor burden in the afatinib and combination treatment groups was significantly less than the tumor burden in the control and pacritinib treatment groups. The combination treatment group displayed the smallest tumor burden (Figure 6A and B and Supplementary Figure S5B).

These above results strongly support the hypothesis that combinatorial inhibition with EGFR and STAT3 inhibitors, as a means of overcoming compensatory survival mechanisms, holds promise as an effective therapeutic strategy for GBM.

## Discussion

Despite advances in our understanding of the aberrant molecular biology underlying GBM, there have been no significant clinical advances and there remains a desperate need for new treatment strategies. The prevalence of *EGFR* mutations in GBM has resulted in a significant focus on EGFR as a potential drug target.^2,3,34^ However, anti-EGFR therapies have failed to improve the progression-free survival of GBM patients.^22,35–37^ This lack of success with EGFR inhibitors may, in part, be due to resistance mechanisms, such as the upregulation of other pro-survival signaling pathways upon EGFR blockade.^8,10,11,13^ A better understanding of the signaling networks and key mechanisms of resistance in GBM, as well as combinations of drugs with effective synergistic modes of action, is crucial for the development of combination regimens. It has been recently shown that EGFR inhibitors trigger adaptive responses through a TNF–JNK–Axl–ERK signaling axis in glioma cells.^36^ In a molecularly heterogeneous disease like GBM, this is likely but one of the many cellular and microenvironment dependent adaptive response mechanisms that occurs in response to EGFR inhibition. Here, we demonstrate that STAT3 signaling is activated in response to EGFR inhibition in both endogenously expressing *EGFR* wt and *EGFRvIII* mt BTSCs grown in vitro under stem cell conditions and in in vivo orthotopic BTSC xenografts. We further demonstrate that a combinatorial strategy, using the brain penetrant and synergistic drugs afatinib and pacritinib, was effective at inhibiting BTSC growth both in vitro and in vivo.

Our in vitro data showed that *EGFR*-mutant BTSCs are more sensitive to EGFR inhibition with afatinib than *EGFR*-wildtype BTSCs. Afatinib accumulated in the serum and the brains of nontumor-bearing SCID mice at micromolar concentrations, indicating effective BBB penetration. Afatinib has been used in the clinic for other cancer types^38–40^ and has demonstrated manageable safety profiles in clinical trials for GBM.^22^ In vivo, afatinib on its own reduced the tumor burden in BT73 and BT147, both highly aggressive *EGFRvIII* mt BTSCs. This work supports the general insights from previous clinical studies,^22^ which suggest that prescreening for *EGFR*-activating mutations may help identify patients who could potentially benefit from anti-EGFR therapy. In a clinical case study, a patient with an *EGFRvIII* mt recurrent GBM was reported to show longer progression-free survival after treatment with afatinib and protracted TMZ.^41^ It is thus likely that there will be a benefit with afatinib for selected patient cohorts with *EGFR-*mutant status. Our results also showed that BTSCs characterized by *EGFR* wt and *PTEN* mt (Supplementary Table S1 and Figure 1) were the most resistant to EGFR inhibition. It has been previously reported that wildtype *PTEN* results in opposed PI3K/AKT signaling and the *EGFR* mutation causes EGFR dependence.^5,42^ Conversely, *PTEN* inactivation results in unopposed PI3K/AKT signaling through other receptor tyrosine kinases.^5,42^ However, we did not find increased p-AKT signaling in BTSCs following treatment with EGFR inhibitors. The differential sensitivities of patient-derived BTSCs to EGFR inhibition further confirm the importance of stratifying patients, based on molecular characteristics, for targeted treatments.

We observed a compensatory increase in pro-survival STAT3 signaling, both in vitro and in vivo following EGFR inhibition. The JAK2/STAT3 pathway is a potent pro-survival signaling pathway and activated STAT3 is expressed at high levels in more than 90% of GBMs^32,43^ and patient-derived BTSCs.^44^ This led us to investigate the effectiveness of a JAK2 inhibitor, pacritinib, in combination with afatinib. We found that, in vitro, concurrent treatment with afatinib and pacritinib was synergistic compared to inhibition of either pathway alone. Importantly, combination treatment also abrogated the sphere-forming frequency of BTSCs. Self-renewal is a key property of cancer stem cells and eliminating this subpopulation of cells is of high relevance for preventing disease relapse.^45,46^

Concurrent administration of afatinib and pacritinib also showed promise in vivo. Strikingly, STAT3 activation in response to EGFR inhibition was abrogated with combinatorial afatinib and pacritinib treatment in vivo. Further, an in vivo assessment of tumor burden revealed a reduction in tumor burden with afatinib and pacritinib compared to the control and single-agent treated animals. The tumors in the combination group were the smallest of any treatment cohort. The reduced tumor burden observed following combinatorial treatment regimens suggests that there would have likely been a survival benefit in the combinatorial arm over the single agent arms of the study. However, there were toxicity issues when co-administering afatinib and pacritinib in vivo. Interestingly, co-delivery of afatinib and pacritinib potentiated the accumulation of pacritinib up to 5-fold in the brain. The increased levels of pacritinib in the brain could help explain the observed toxicity in the combinatorial treatment group and needs to be further investigated. In future animal studies, it will be useful to determine the best dose combinations for the 2 drugs and the most effective delivery schedule to achieve efficacy while limiting toxicity. We have previously reported that pacritinib displays more favorable pharmacokinetic properties in humans than in mice.^18^ Pacritinib has completed a phase III trial for myelofibrosis, with daily oral dosing, and has demonstrated promising pharmacokinetic and safety profiles with limited toxicities in humans.^38^ It would also be of high clinical relevance to further investigate other JAK/STAT3 inhibitors, existing or currently under development, in combinatorial studies with brain-penetrant EGFR inhibitors such as afatinib. For example, the JAK2/STAT3 inhibitor, WP1066, which we previously investigated in BTSC orthotopic xenograft models,^17^ and found here to be synergistic with afatinib, is especially promising and was recently granted approval by the FDA for GBM as an orphan drug.

Administering drug combinations to cancer patients has the potential for better clinical outcomes compared to individual agents, where mechanisms of resistance are often observed. When synergism is found with 2 drugs, there is an added benefit of using lower drug doses to help minimize drug resistance as well as drug toxicities (reviewed in Ref. ^47^). The results in this study suggest that afatinib and pacritinib are promising therapeutic drugs with synergistic benefit and may be considered for combinatorial therapy for GBM. Given that both EGFR and STAT3 are crucial signaling hubs in GBM, dual targeting may provide a survival advantage that monotherapies have failed to offer. In particular, the data shown here demonstrate that EGFR inhibition may still be efficacious for selected cohorts of *EGFR*-mutant GBM patients. However, therapeutic resistance is likely to arise following EGFR inhibition in such cohorts as well. Thus, clinical strategies combining EGFR inhibition with therapeutic approaches aimed at targeting pro-oncogenic STAT3 signaling hold promise for GBM treatment.

## Funding

This research project was supported in part by the Canadian Institutes of Health Research grants (to H.A.L. and S.W.) and graduate scholarship (to K.V.J.), CTI BioPharma Corp. (to H.A.L. and S.W.), and the Alberta Cancer Foundation (scholarship to K.V.J.). S.W. is also supported by SU2C Canada Cancer Stem Cell Dream Team research funding (SU2C-AACR-DT-19-15) provided by the Government of Canada through Genome Canada and the Canadian Institutes of Health Research.

## Supplementary Material

vdaa020_suppl_Supplementary_Material

vdaa020_suppl_Supplementary_Legends

## Notes

**Authorship.** Conceptualization: K.V.J., H.A.L., and S.W. Methodology: K.V.J., A.A., and X.H. Investigation: K.V.J. Formal Analysis and Visualization: K.V.J., A.A., and X.H. Writing: K.V.J. and H.A.L. Writing—Review and Editing: K.V.J., H.A.L., and S.W. Funding Acquisition: H.A.L. and S.W. Supervision: H.A.L. and S.W.
