# Supplementary material for: EGFR blockade in GBM brain tumor stem cells synergizes with JAK2/STAT3 pathway inhibition to abrogate compensatory mechanisms in vitro and in vivo
Source: Neurooncol Adv. 2020 Feb 18;2(1):vdaa020. doi: 10.1093/noajnl/vdaa020 (PMC7086303; doi:10.1093/noajnl/vdaa020)
Supplement: vdaa020_suppl_Supplementary_Material [file vdaa020_suppl_supplementary_material.pdf]

Supplemental Table 1: Identification of a diverse panel of GBM BTICs representative of the different mutational statuses of GBM patients.

**BTIC Line**	**Diagnosis**	**Age**	**Sex**	**MGMT status**	**EGFR status**	**P53 status**	**PTEN status**	***IDH1* status**	***NF1* status**	***CDKN2A* status**
**BT12**	GBM-r	59	M	U	wt	wt	mut	wt	N/A	N/A
**BT48**	GBM	68	M	M	mut	wt	mut	wt	wt	homo del
**BT50**	GBM	61	M	M	wt	wt	mut	wt	wt	homo del
**BT53**	GBM	59	M	M	mut	mut	wt	wt	wt	homo del
**BT67**	GBM	44	M	M	wt	wt	mut	wt	mut/het del	homo del
**BT68**	GBM	59	M	U	vIII	wt	wt	wt	N/A	N/A
**BT69**	GBM	50	M	U/M	het	mut	mut	wt	N/A	homo del
**BT73**	GBM	52	M	M	vIII	mut	mut	wt	N/A	homo del
**BT124**	GBM	N/A	N/A	U	wt	mut	mut	wt	N/A	N/A
**BT143**	GBM-r	46	F	M	wt	mut	mut	wt	mut	homo del
**BT147**	GBM-r	56	M	U	vIII	mut	mut	wt	wt	homo del
**BT189**	GBM	55	F	U	wt	mut	mut	wt	wt	wt

Common GBM molecular alterations including *MGMT* promoter methylation*, EGFR*, *PTEN*, *TP53*, *NF1*, *IDH1*, and *CDKN2A* mutation statuses for the 11 BTIC lines used in this study. Mut indicates mutant and wt indicates wild-type. U indicates unmethylated and M indicated methylated. U/M indicates hemi-methylation. vIII indicates *EGFR* variant III, an activating deletion characteristic of GBM, het del indicates a heterozygous deletion, homo del indicates a homozygous deletion, N/A indicates not available.

(A)

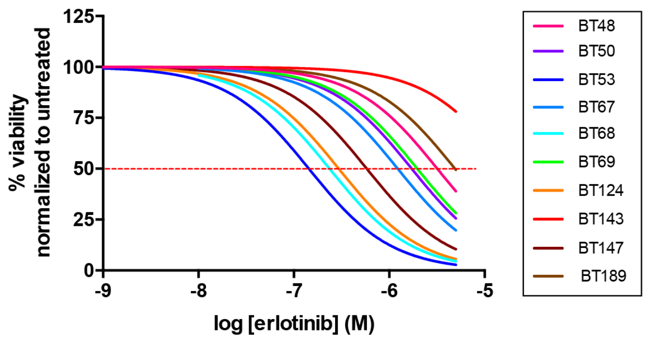

BTIC line	Erlotinib (μM)	EGFR status
**BT48**	3.2	G598V
**BT50**	17.8	wt
**BT53**	0.14	G598V
**BT67**	1.2	wt
**BT68**	0.24	vIII
**BT69**	1.9	het
**BT124**	0.29	wt
**BT143**	1.7	wt
**BT147**	0.58	vIII
**BT189**	4.9	wt

(B)

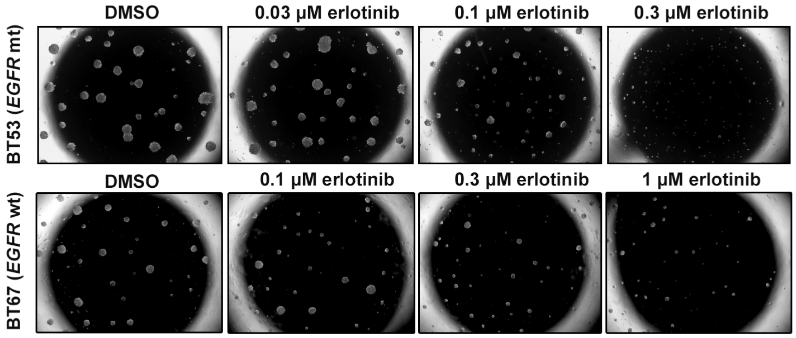

(C)

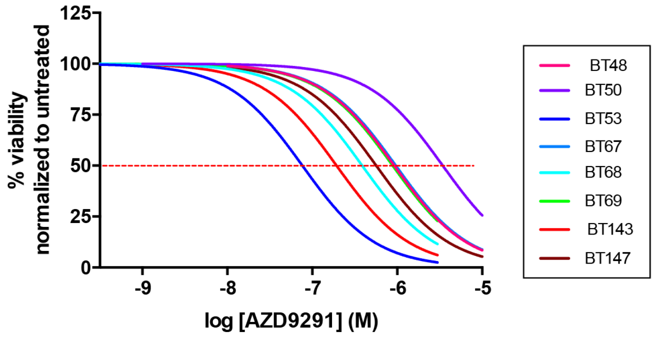

BTIC line	AZD9291 (μM)	EGFR status
**BT48**	0.92	G598V
**BT50**	3.4	wt
**BT53**	0.076	G598V
**BT67**	0.96	wt
**BT68**	0.39	vIII
**BT69**	0.88	het
**BT143**	0.193	wt
**BT147**	0.57	vIII

(D)

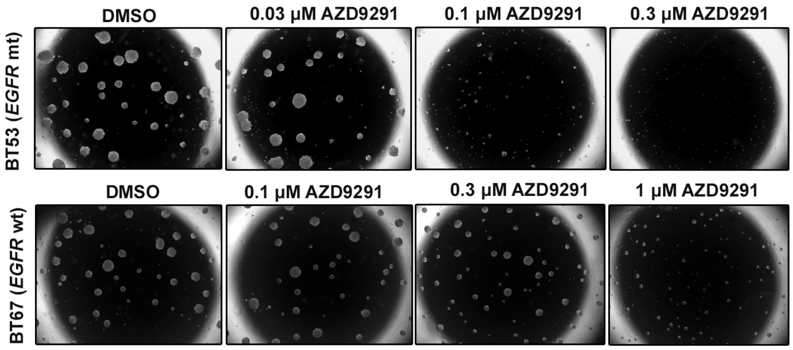

(E)

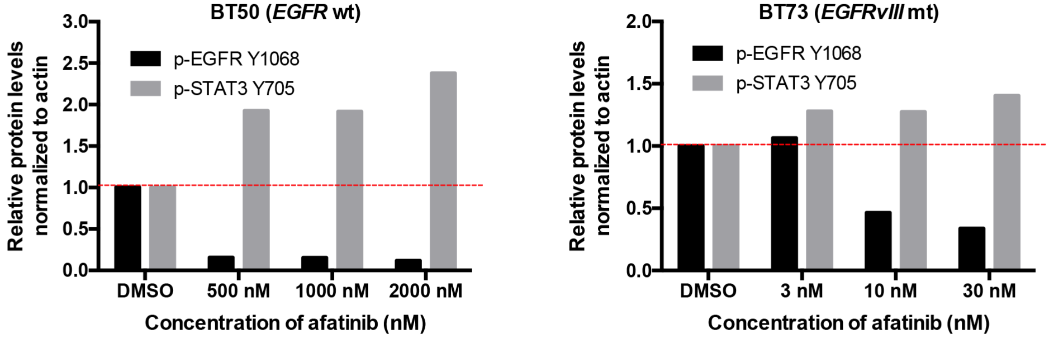

Supplemental Fig. 1

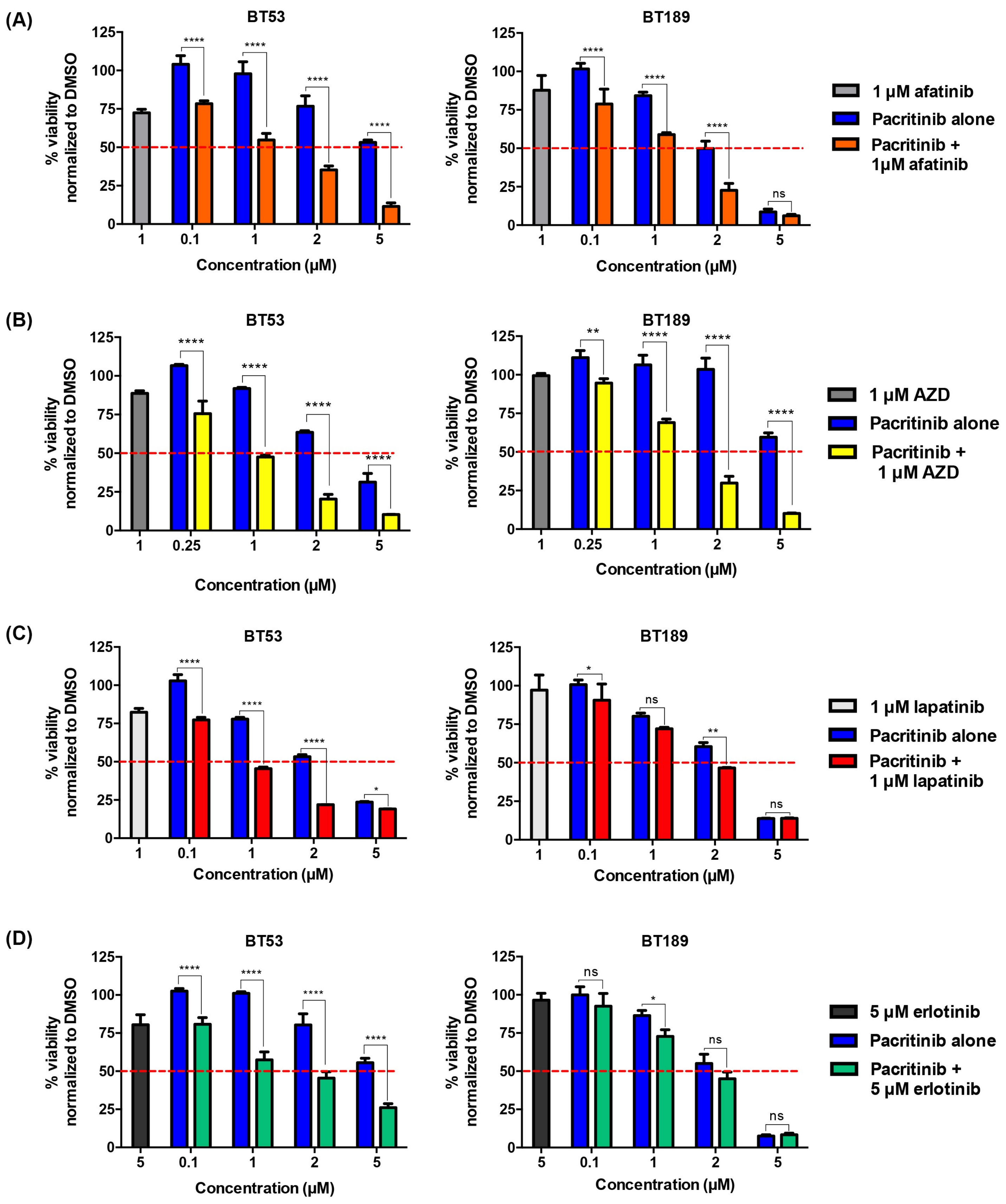

Supplemental Fig. 2

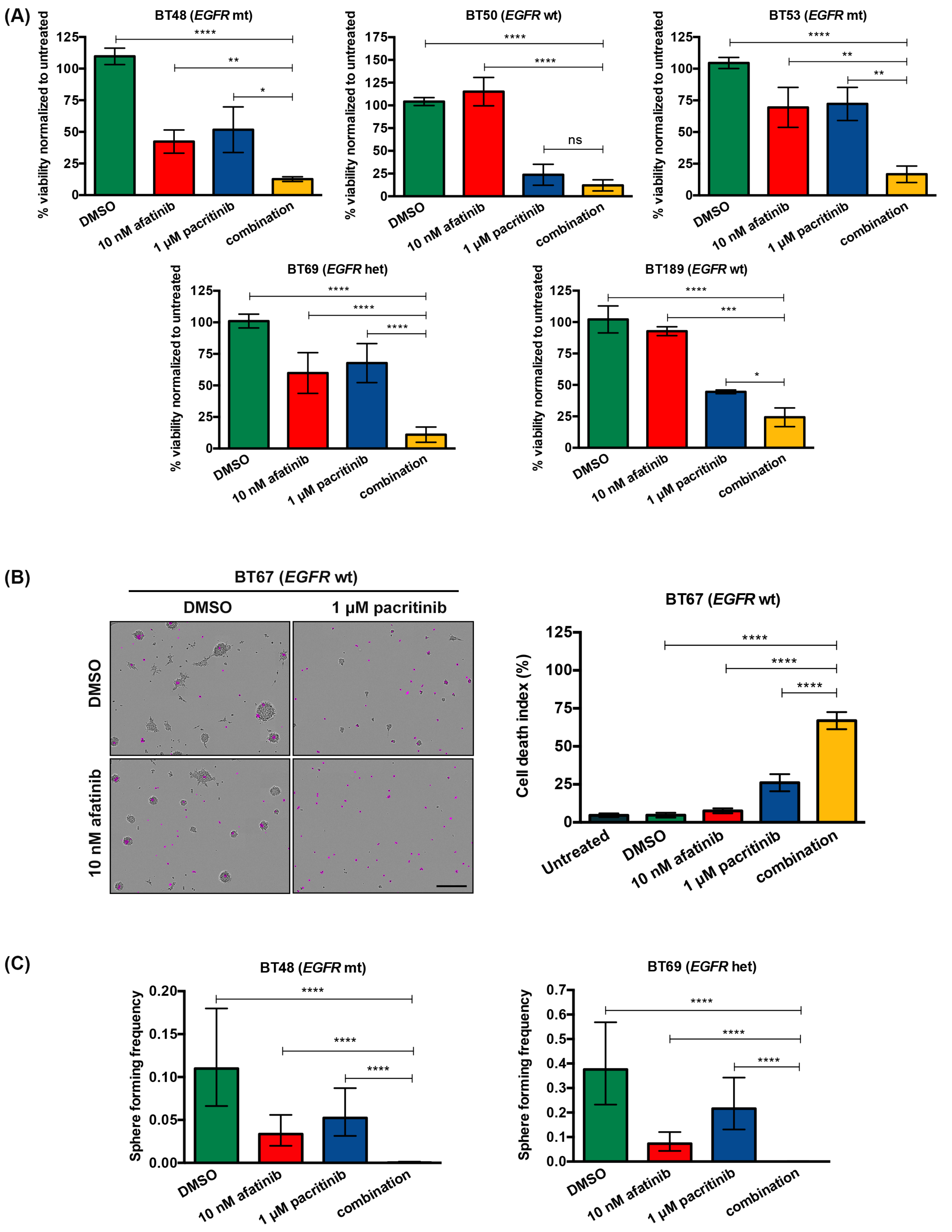

Supplemental Fig. 3

(A)

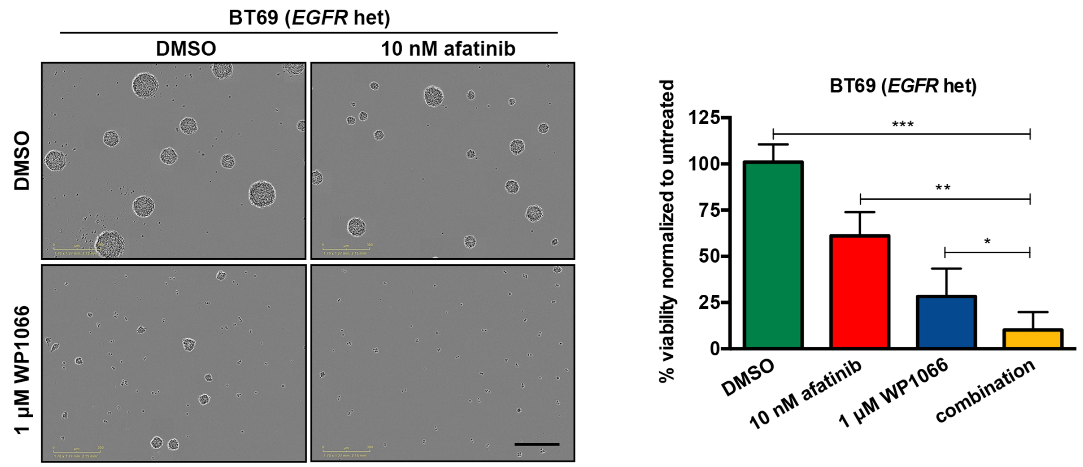

(B)

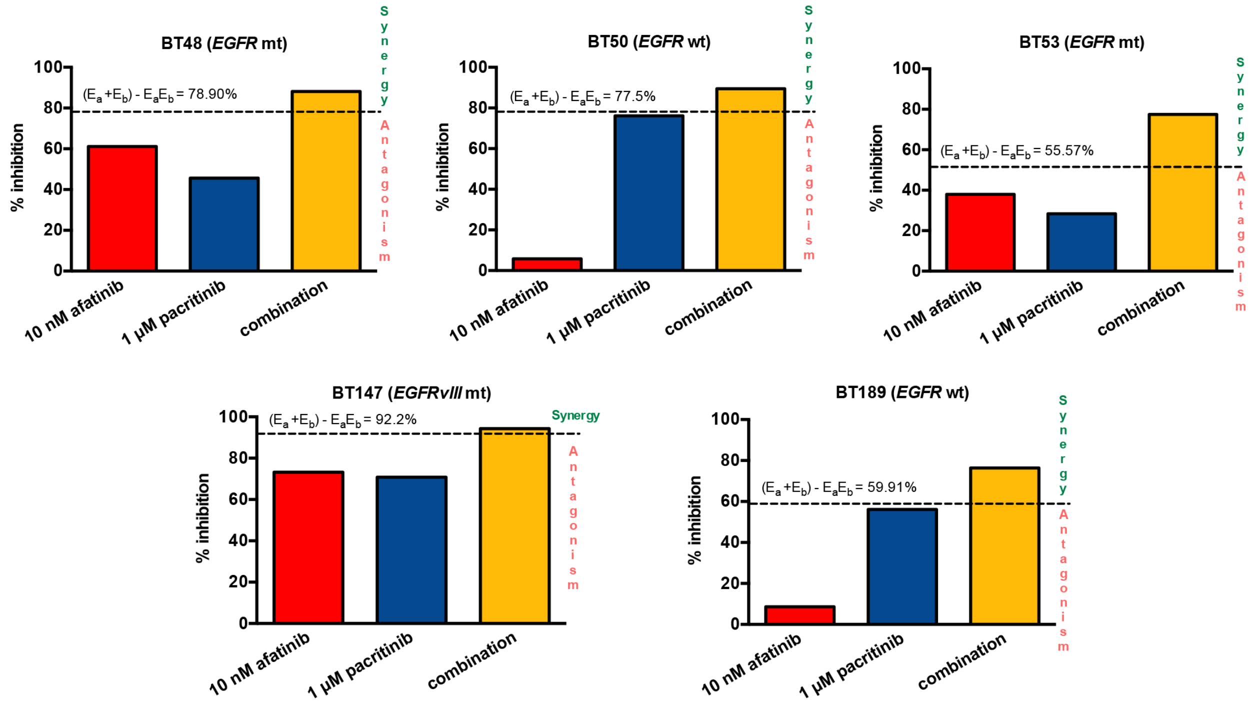

(C)

BT69 (*EGFR* het)
erlotinib (µM)

% inhibition pacritinib (µM)	10	5	3	1	0
1	85.0022841	80.3849223	78.2738674	70.8339955	-5.1306994
0.3	77.5243282	65.4335772	59.6033463	51.4351889	-14.877944
0.1	76.5056222	59.7428594	54.6965544	42.1872791	-8.7372337
0	68.7835636	58.7946963	50.6916304	38.5794293	0

erlotinib (µM)

Excess over bliss pacritinib (µM)	10	5	3	1	0
1	17.820342	23.7043463	30.1121012	35.4058712	0
0.3	13.3851286	12.7693831	16.2477875	21.9938779	0
0.1	10.4495115	4.54836681	8.3131114	8.9743086	0
0	0	0	0	0	0

BT147 (*EGFRvIII* mt)
erlotinib (µM)

% inhibition pacritinib (µM)	10	5	3	1	0
1	87.0287556	85.5860001	84.8352194	84.9468128	38.5645045
0.3	76.7758572	74.9831207	70.8651323	67.2372072	-11.39176
0.1	76.7923093	71.3976478	67.3668418	61.2299209	-5.7182979
0	76.0670927	75.2702882	66.7276967	64.7736307	0

erlotinib (µM)

Excess over bliss pacritinib (µM)	10	5	3	1	0
1	1.73205579	0.77882109	5.27622379	6.58830734	0
0.3	3.43514385	2.52998203	7.92773668	6.4764801	0
0.1	2.09377154	-2.4585218	2.54175454	-1.529361	0
0	0	0	0	0	0

Supplemental Fig. 4

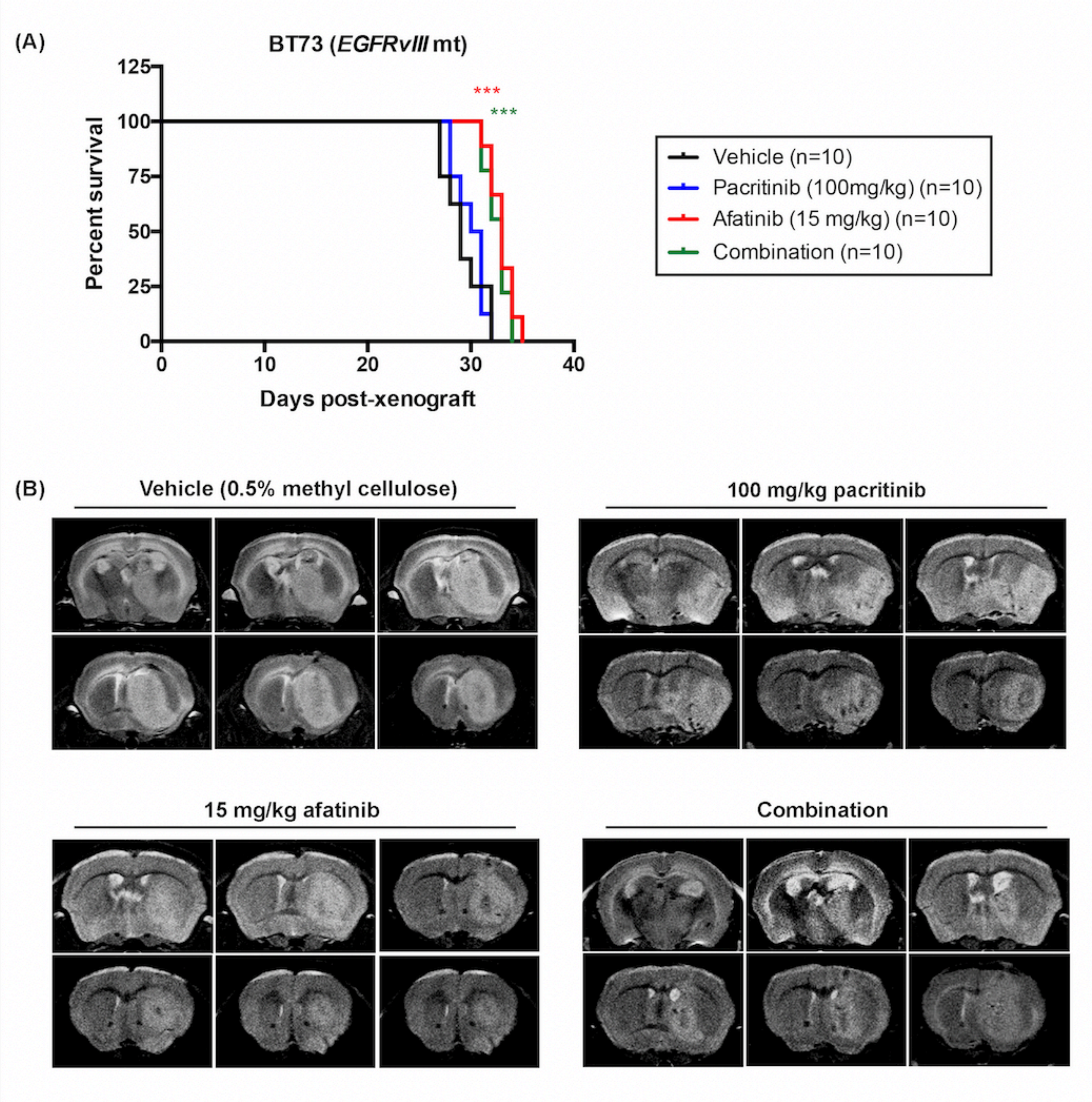

Supplemental Fig. 5
