# Supplementary material for: EGFR blockade in GBM brain tumor stem cells synergizes with JAK2/STAT3 pathway inhibition to abrogate compensatory mechanisms in vitro and in vivo
Source: Neurooncol Adv. 2020 Feb 18;2(1):vdaa020. doi: 10.1093/noajnl/vdaa020 (PMC7086303; doi:10.1093/noajnl/vdaa020)
Supplement: vdaa020_suppl_Supplementary_Legends [file vdaa020_suppl_supplementary_legends.docx]

**Supplemental Legends**

**Table S1. Identification of a diverse panel of GBM BTSCs representative of the different mutational statuses of GBM patients.** Common GBM molecular alterations including MGMT promoter methylation, *EGFR, PTEN, TP53, NF1, IDH1,* and *CDKN2A* mutation statuses for the twelve BTSCs used in this study. Mut indicates mutant and wt indicates wild-type. U indicates unmethylated and M indicated methylated. U/M indicates hemi-methylation. vIII indicates EGFR variant III, an activating deletion characteristic of GBM, het del indicates a heterozygous deletion, homo del indicates a homozygous deletion, N/A indicates not available.

**Fig. S1:** **EGFR inhibition with erlotinib, AZD9291, and afatinib, effectively decreases BTSC viability and sphere forming capacity and has on-target activity on phospho-EGFR.** (A) Erlotinib dramatically decreased cell viability in a dose dependent manner at micromolar concentrations in a panel of molecularly diverse BTSCs. The IC_50_ values ranged from 0.14 μM to 17.8 μM. (B) Representative images of BT53 (*EGFRvIII* mt) and BT67 (*EGFR* wt) spheres following erlotinib treatment. (C) AZD9291 dramatically decreased cell viability in a dose dependent manner at micromolar concentrations in a panel of molecularly diverse BTSCs. The IC_50_ values ranged from 0.076 μM to 3.4 μM. (D) Representative images of BT53 (*EGFRvIII* mt) and BT67 (*EGFR* wt) spheres following AZD9291 treatment. (E) Quantification of representative blots. Treatment of BT50 (*EGFR* wt) and BT73 (*EGFRvIII* mt) with afatinib for 3 hours markedly reduced phosphorylation of EGFR on tyrosine 1068 (p-EGFR Y1068). Upon EGFR inhibition, there was an activation of STAT3 as seen by an increase in the phosphorylation of STAT3 on tyrosine 705. Relative protein levels of p-EGFR Y1068 and p-STAT3 Y705, normalized to actin, are quantified in the bar graph.

**Fig. S2:** **48-hour screen using four EGFR inhibitors in combination with the JAK2/STAT3 inhibitor pacritinib**. All the EGFR inhibitors tested displayed efficacy when combined with pacritinib (* denotes P < 0.05, ** denotes P < 0.01, *** denotes P < 0.001, and **** denotes P < 0.0001 vs DMSO; Sidak’s multiple comparison two-way ANOVA).

**Fig. S3: Combined inhibition of the EGFR and JAK2/STAT3 pathways is more effective than the inhibition of either pathway alone.** (A) Combined treatment with afatinib and pacritinib decreased alamarBlue conversion in five BTSC cultures (* denotes P < 0.05, ** denotes P < 0.01, *** denotes P < 0.001, and **** denotes P < 0.0001 vs DMSO; Sidak’s multiple comparison two-way ANOVA). (B) Representative images for BT67 (*EGFR* wt) of cell death index. Scale bars represent 300 μm. (C) Limiting dilution analysis demonstrated that combined inhibition caused a significant decrease in self-renewing frequency in the BTSCs compared to the decrease that was observed when inhibiting a single pathway (**** denotes P < 0.0001).

**Fig. S4:** **Combined inhibition of the EGFR and JAK2/STAT3 pathways is synergistic in BTSCs.** (A) Combined treatment with afatinib and the JAK2/STAT3 inhibitor WP1066 decreased sphere formation and alamarBlue conversion (representative BTSC BT69 (*EGFR* het) shown) (* denotes P < 0.05, ** denotes P < 0.01, and *** denotes P < 0.001 vs DMSO; Sidak’s multiple comparison two-way ANOVA). The scale bar represents 300 μm. (B) Combined treatment with suboptimal doses of afatinib and pacritinib was synergistic in all BTSCs tested. Bliss independence shows extensive synergy with these suboptimal concentrations. (C) Combined inhibition with erlotinib and pacritinib is synergistic in BTSCs. Percent inhibition and excess over bliss values are shown for two representation BTSCs, BT69 (*EGFR* het) and BT147 (*EGFRvIII* mt). An excess over Bliss > 0, indicates synergy. Excess over Bliss = 0, indicates additive. Excess over Bliss < 0 indicates less than additive.

**Fig. S5: Combinatorial treatment is effective at decreasing tumor burden, despite toxicities preventing a survival benefit**. (A) The afatinib and combination arms show a survival benefit over the control and pacritinib only arms; however, the combination arm failed to provide further benefit over the afatinib arm due to systemic toxicities. (B) Representative serial 0.5 mm sections for each of the treatment arms. Mice were imaged at the 3-week time point.
